# Leveraging Routine Blood Tests, Plasma pTau‐217, and Machine Learning for Enhanced Detection and Monitoring of Preclinical Alzheimer's Disease

**DOI:** 10.1002/alz70860_097106

**Published:** 2025-12-23

**Authors:** Liwei Ma, Yihan Wang, Chenyin Chu, Andrew Huynh, Georgios Zisis, Colin L Masters, Benjamin Goudey, Liang Jin, Yijun Pan

**Affiliations:** ^1^ University of Melbourne, Parkville, VIC, Australia; ^2^ Florey Institute of Neuroscience and Mental Health, Parkville, VIC, Australia; ^3^ Austin Health, Melbourne, VIC, Australia; ^4^ The Florey Institute of Neuroscience and Mental Health, The University of Melbourne, Parkville, Melbourne, VIC, Australia

## Abstract

**Background:**

Preclinical Alzheimer's disease (AD) develops 15–20 years before symptom onset. This study aimed to develop models to enhance the detection and monitoring of preclinical AD.

**Method:**

A retrospective cohort study was conducted based on data from the A4 study, spanning February 28, 2014, to June 8, 2023. A total of 420 A4 participants with preclinical AD were analyzed to examine associations between hematology/serum biochemistry markers and brain amyloid‐PET SUVR or plasma pTau‐217 levels. Multivariable linear regression models were used for these analyses, and machine learning models were developed to predict these AD biomarkers over a 4.5‐year period.

**Result:**

Increased monocyte count was associated with elevated brain amyloid‐PET SUVR, while higher mean corpuscular volume (MCV) was linked to elevated plasma pTau‐217 levels. Additionally, elevated AST‐to‐ALT ratios and creatinine levels were associated with increased amyloid‐PET SUVR and plasma pTau‐217 levels. Hematology and serum biochemistry markers notably improved the predictive performance of models for plasma pTau‐217 levels. However, monocyte count was not a strong predictor of amyloid‐PET SUVR.

**Conclusion:**

The machine learning models demonstrated promising potential for predicting AD biomarkers, emphasizing the need for future research to evaluate their applicability in both clinical and research contexts.

